# The Clinical Trial Outcomes of Cranberry, D-Mannose and NSAIDs in the Prevention or Management of Uncomplicated Urinary Tract Infections in Women: A Systematic Review

**DOI:** 10.3390/pathogens11121471

**Published:** 2022-12-05

**Authors:** Jenane Konesan, Lu Liu, Kylie J. Mansfield

**Affiliations:** 1 School of Biomedical Sciences, UNSW, Sydney, NSW 2052, Australia; 2 Graduate School of Medicine, University of Wollongong, 2522 Wollongong, Sydney, NSW 2052, Australia

**Keywords:** urinary tract infection, cranberry, mannose, non-steroidal anti-inflammatory, prevention, treatment

## Abstract

The use of antibiotics in the treatment of UTIs is contributing to resistance. Hence, the outcome of human clinical trials of nonantibiotic remedies for preventing or treating UTI is of significant interest. This systematic review aimed to identify, summarise and evaluate the evidence for the outcomes of different nonantibiotic options including cranberry, D-mannose and non-steroidal anti-inflammatory drugs (NSAIDs). PubMed, Embase and Scopus were searched for manuscripts relating to nonantibiotic treatment of UTI including cranberry, mannose and NSAIDs. After title and abstract screening, data were extracted from 21 papers that were published in English and related to the treatment or prevention of uncomplicated UTI in adult women. We identified twelve papers examining the effects of cranberry, two papers examining D-mannose, two papers examining combination treatments (cranberry and D-mannose) and five manuscripts investigating the effects of NSAIDs. There is low-level evidence, from a small number of studies, supporting the use of D-mannose or combination treatments for potentially preventing UTIs in adult women without producing burdening side effects. However, larger and more randomised double-blinded trials are needed to confirm this. In comparison, the multiple studies of cranberry and NSAIDs produced conflicting evidence regarding their effectiveness.

## 1. Introduction

Urinary tract infections (UTIs) are most common in women, comprising nearly 25% of all infections [1,2]. Approximately 50 to 60% of women will report a UTI at least once in their lifetime, with one in three women experiencing a symptomatic UTI treated with antibiotics by age 24 [3]. Of these women, 20–40% will experience a recurrent UTI (r-UTI) [4], which is defined as two UTI episodes in 6 months or three in 12 months [5]. Recurrent UTIs typically occur within three months of the first infection, even with a complete symptomatic resolution by using first-line antimicrobial therapy (antibiotics). There are different risk factors that predispose women of different age groups to experience UTIs. In pre-menopausal women, maternal history of UTIs, sexual intercourse, changes in bacterial flora and use of spermicide contraception can increase their chances of experiencing UTIs [6]. Post-menopausal women typically experience a decline in oestrogen levels and an onset of menopausal symptoms that increase the likelihood of UTIs, likely due to changes in the vaginal bacterial flora [7]. Common UTI symptoms include dysuria, urinary frequency, urgency, suprapubic pain, possible haematuria and cloudy urine with an unpleasant odour [8].

**Figure 1:**
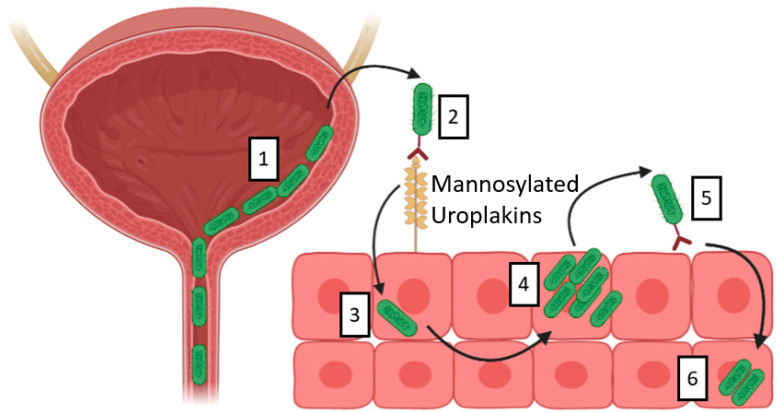
Proposed model of UPEC-induced UTI. UPEC is thought to ascend the urethra (1) and then bind to mannose residues on uroplakins on the urothelial cell surface (2). Following binding, UPEC is internalised (3). After internalisation UPEC can follow two pathways, either UPEC multiplies within the urothelial cells (4) and then effluxes out to recolonise the bladder (5); or small numbers of UPEC remains in a quiescent state within the host urothelial cells (6) [17].

The most common causative agent of UTIs is the gram-negative bacteria uropathogenic *Escherichia coli* (*UPEC*), accounting for nearly 80% of all UTIs [9]. UPEC is thought to ascend the urethra before adhering to the urothelium and colonising the urinary bladder (Figure 1). Animal models of acute cystitis have demonstrated that binding of UPEC to the urothelium leads to invasion, rapid replication and the formation of intracellular bacterial communities (IBCs) [10]. The endpoint of this pathway is defined by UPEC fluxing out of infected or exfoliated superficial urothelial cells and is associated with the presence of long filamentous bacterial cells [11]. Intracellular bacterial communities have also been observed in urothelial cells isolated from women and children with UTI [12,13,14] and women with LUTS [15] and urge incontinence [16]. Additionally, filamentous bacteria have been observed in the urine of women with acute cystitis [12].

To treat UTIs, nitrofurantoin or trimethoprim–sulfamethoxazole (TMP-SMX) is typically prescribed as first-line antimicrobial therapy for uncomplicated UTIs. However, in many countries, antibiotic resistance has significantly increased towards these first-line antimicrobial drugs [18]. This has led to multi- and pan-drug resistant strains, thus imposing a significant burden on the economy and healthcare system. This significant increase in antibiotic resistance has burdened the Australian healthcare system with costs over AUD 900 million and estimated to reach AUD 1.6 billion by 2030 [19]. Recently, a study demonstrated the persistence of UPEC in the face of several different antibiotic treatments [20]. As a consequence of the continuously diminishing efficacy of antibiotics, new strategies and alternative treatments have been employed for UTI patients [21]. A short-term approach has involved tightening antimicrobial stewardship within medical practises and delegating antibiotics more judiciously to patients [22].

In recent years, there has been significant interest in utilising nonantibiotic remedies that either prevent or treat UTIs. The most common nonantibiotic agents are cranberry products, D-mannose or non-steroidal anti-inflammatory drugs (NSAIDs) [23]. Cranberry products are derived from the cranberry fruit, also called vaccinium macrocarpon, which was historically consumed by North American Indians to treat UTIs [24]. Fruits within the same family are the European cranberry (*V. oxycoccos*), lingonberry (*V. vitisidaea*) and blueberry (*V. myrtillus*) [25]. Cranberry contains an array of active constituents, but a number of studies have suggested that proanthocyanidins (PAC) and fructose are the main active ingredients to prevent bacterial adhesion to the urinary tract [26].

Another nonantibiotic product that may have the potential to prevent or treat UTIs is D-mannose. Like cranberry products, D-mannose acts to block bacterial adhesion to urothelial cells [27]. It has been proposed that D-mannose has a similar structure to the usual bacterial binding site on the uroplakins that line the urothelium. Hence, a sufficiently high concentration of D-mannose can cause saturation of bacterial adhesins and prevent bacteria from binding to the urinary tract [28].

The third nonantibiotic agent that has been used in treating symptomatic UTI is NSAIDs. When bacteria bind to or invade the urinary tract, they trigger an inflammatory response. This causes the symptoms of pain and frequency associated with UTIs. Therefore, NSAIDs could be another antibiotic-sparing treatment to provide symptomatic relief from inflammation-associated symptoms of UTI [29]. Experimental evidence demonstrates that NSAIDs inhibit the inflammatory response at doses equivalent to that effective in improving bladder functions [30]. However, NSAIDs do not affect bacterial growth and have no impact on bacterial attachment. Hence, the effects of NSAIDs are likely mediated through their anti-inflammatory properties rather than direct interactions with uropathogens in the bladder [31]. Therefore, NSAIDs may appear as an attractive alternative for those experiencing painful bouts of symptomatic UTIs.

The literature evidence, therefore, suggests that cranberry and D-mannose may be suitable for preventing bacterial adhesion to the urinary tract, and these agents may be useful as prophylaxis to prevent UTIs. In contrast, NSAIDs are better used for the symptomatic treatment of uncomplicated bacterial cystitis. There have been several previously published reviews, with or without meta-analyses, on the effectiveness of individual nonantibiotic agents including, cranberry [32,33,34,35], D-mannose [36,37,38] and NSAIDs [39,40] in preventing or treating UTIs. However, this review is distinct in applying more restrictive inclusion criteria to the comparison of the effectiveness of three different nonantibiotic agents in women over eighteen with uncomplicated UTIs (cystitis). The purpose of this review is to evaluate and critically appraise the design and outcomes of clinical trials of nonantibiotic products, including cranberry, D-mannose and NSAIDs, in either preventing or treating uncomplicated cystitis in otherwise healthy adult women.

## 2. Results and Discussion

### 2.1. Search Results

**Figure 2:**
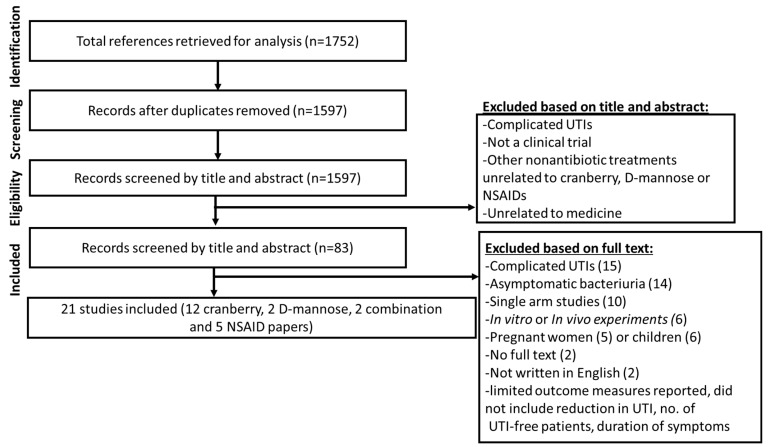
PRISMA flowchart outlining the protocol used in this systematic review. Eligible studies were clinical trials conducted in humans, undertaken with a focus on the prevention or treatment of UTIs using three nonantibiotic agents (cranberry, D-mannose and NSAIDs). Trials were included if they focused on the prevention or treatment of uncomplicated UTI (cystitis) in adult women (≥18 years old). Studies that focused on UTI in children or pregnant women were excluded as were studies focused on asymptomatic bacteriuria. In vivo or in vitro studies conducted in animal or cell culture models of UTI were also excluded.

A total of 1752 references were initially retrieved. After eliminating duplicates and those outside inclusion criteria (refer to the Methods and Materials section for details), data were extracted from twenty-one papers in this review, with twelve papers for cranberry, two papers for D-mannose, two combination treatments (cranberry and D-mannose) and five for NSAIDs. A PRISMA flow chart of the search results is shown in Figure 2.

### 2.2. Clinical Trial Designs

The optimal approach for the design of a clinical trial is to include a randomised, controlled trial [41]. Of the 21 studies included in this review 18 (81%) were described as randomised controlled trials, with 13 (59%) being double-blind and 8 (36%) including a placebo or control group (Table 1). In most of the studies examined as part of this SLR, woman participants were between their early 30s to mid-50s (Table 1). Only two of these trials studied women in their early to mid-20s, which were from the cranberry trial A8 [42] and A9 [43]. Hence, the age range of the women included in these trials of nonantibiotic agents is an appropriate population to survey and the results are therefore generalisable to those who are likely to experience uncomplicated UTIs.

In this review, the majority of the studies examined the effectiveness of nonantibiotic agents in the prevention of UTIs. This was particularly true for the studies examining the effectiveness of cranberry or D-mannose. Of the 16 trials examining the prevention of UTI, (Table 1A–C), all except one study, A10 [33], examined the preventative effect of cranberry and D-mannose. In contrast, the five trials examining the effectiveness of NSAIDs all investigated NSAIDs as a treatment of acute cystitis (Table 1D).

**Figure 3:**
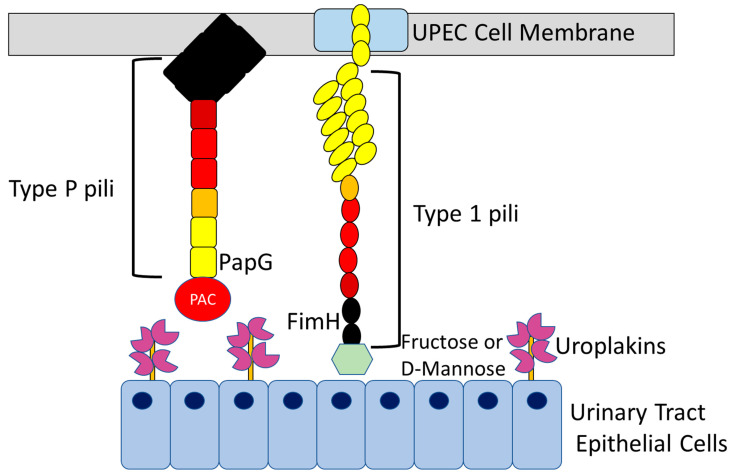
Inhibition of UPEC adhesion molecules by cranberry active ingredients (PAC and fructose) and D-mannose. UPEC associated with UTI commonly expresses two adhesion molecules Type P and Type 1 pilli. Type P pilli contain the adhesin molecule PapG on the tip. Proanthocyanidin (PAC) binds to this molecule and inhibits UPEC adhesion to the epithelial cells that line the urinary tract. Type 1 pilus ends with a FimH adhesin molecule at the tip which usually binds to highly N-mannosylated uroplakin proteins which are abundantly expressed on the apical surface of the urothelium. Fructose and D-mannose bind to the FimH residues thus reducing bacterial adhesion to the urothelium.

This approach to the study design matches the known actions of these agents. The two main active ingredients in cranberry are PAC [26,60] and fructose [61] which are both thought to be involved in the inhibition of *E. coli* adherence to the urinary bladder (Figure 1). In vitro, fructose has been shown to inhibit type 1 fimbriae adherence [61] and PAC to inhibit the p-fimbriae [60] (Figure 3). UPEC expresses a number of adhesion molecules, which are fimbrial adhesin or pili. These hair-like proteinaceous structures project outwards from the bacterial cell membrane [62]. There are many different *E. coli* adhesins including type 1, type 3, type 9, S, P, F1C and Auf [63]. However, the type 1 and P pilus of UPEC are the most prevalent adhesion factors present in strains known to cause UTI [64]. 

Type 1 pilus ends with a FimH adhesin molecule at the tip [65]. FimH commonly binds to the highly N-mannosylated glycan of the uroplakin protein UP1a [66] which is abundantly expressed along the apical surface of the urothelium aiding the adhesion of UPEC to the bladder [66]. Fructose [61] and D-mannose [27] are thought to bind to the FimH residues thus reducing bacterial adhesion to the urothelium (Figure 3). Laboratory studies have shown that fructose induces a 15-fold reduction in affinity to Fim H compared to D-mannose [67].

Type P fimbriae are generally thought to be involved in UPEC adhesion in upper urinary tract infections or pyelonephritis [68]. In the case of type P fimbriae, the tip adhesin molecule PapG binds to PAC (Figure 3) which is postulated to mediate the inhibition of UPEC adhesion in the presence of cranberry [69]. PAC binding to PapG inhibits type P pili from interacting with the glycolipid receptors on renal epithelial cells [70]. It is interesting to note that the majority of the trials included in this study have reported the concentration of PAC in their active treatment but have not considered the fructose concentration. This is especially important as the type P fimbriae, to which PAC binds are generally thought to be responsible for initiating upper UTIs. However, one study has postulated a reduction in bacterial expression against type 1 pili following treatment with PAC [71]. 

As mentioned above, D-mannose, similar to fructose, has been shown to bind to the FimH adhesions of type 1 pili and block bacterial adhesion to urothelial cells (Figure 3) [27,72,73]. This may be because D-mannose has a similar structure to the binding site of these mannosylated uroplakins along the urothelium. Hence, a sufficient concentration of D-mannose can cause saturation of FimH adhesins and prevent bacterial adhesion [74] to the urothelium. D-mannose was shown to significantly reduce bacteriuria in rats with an efficacy dependent upon the concentration of both mannose and bacteria [28].

In contrast to cranberry and D-mannose preventing UTI, NSAIDs are used for the symptomatic treatment of acute cystitis (Table 1D). This is because when bacteria bind to or invade the urinary bladder, it triggers an inflammatory response in the host. This leads to prostaglandin production in conjunction with elevated levels of specific cytokines (IL-5, IL-6, IL-8) and neutrophil migration to the site of injury [75]. As a result, this inflammatory response causes the symptoms of pain and frequency commonly associated with UTIs [29]. Therefore, NSAIDs could be another antibiotic-sparing treatment to provide symptomatic relief from inflammation-associated symptoms of UTIs [29]. Research suggests that the NSAID, ibuprofen, masks the symptoms, thus allowing the host’s immune system to clear the infection independent of antibiotics [76].

Recurrent UTI is defined as two episodes of UTI in 6 months or three UTI episodes in 12 months [5]. Therefore, studies examining the effectiveness of nonantibiotic treatments on the prevention of recurrent UTIs were of longer duration (6 to 12 months, Table 1A–C). As a consequence, the 2-month period of the cranberry trial described in trial A5 [47] may not be applicable long-term compared to the other trials evaluating the prevention of recurrent UTI. Similarly, trial A10 [33] was unusual amongst the trials of cranberry as it had only a 7-day course (Table 1A). Most of the studies on the effectiveness of cranberry ranged from 5.5 months to a year (Table 1A). However, for D-mannose, both trials went on for 6 months (Table 1B). Combination trials ranged from 3 months to a year (Table 1C). While those studies evaluating the symptomatic treatment of UTIs in women with NSAIDS tended to be of short duration (3 to 5 days, Table 1D) due to the short duration of each episode of UTI.

### 2.3. Interventions, Active Ingredients and Comparisons

As can be seen from Figure 2, both cranberry and D-mannose prevent UTIs by binding to adhesion molecules located on the bacterial cell membranes. The effectiveness of this binding in preventing UTI is dependent on the concentration of the agent, either cranberry or D-mannose, relative to the concentration of bacteria. This systematic review of the literature identified that there was great variability in the concentration of both cranberry and D-mannose in the trials examined.

Cranberry trials used either a capsule (A1 [41], A3 [45], A4 [46], A5 [47], A10 [33], A11 [50] and A12 [51]) or juice (A2 [44], A6 [48], A7 [49], A8 [42] and A9 [43]) to administer the active ingredients to their patients (Table 1A). Regarding the cranberry control group, all except trials A6 [48], A10 [33], A11 [50] and A12 [51] used a placebo. In these cases, trial A6 [48] used a lactobacillus drink and no intervention as a comparison. Trials A10 [33] and A11 [50] utilised antibiotics and A12 [51] used a lower dose of cranberry for the control group. A few trials utilised a treatment that contained cranberry and other nonantibiotic products, perhaps to generate a synergistic effect in eliciting an anti-adherence effect (Table 1A). This includes using BKProCyan (A1, [41]), which combined lactobacilli and cranberry extract or utilising a capsule containing cranberry, propolis and zinc (A3 [45]) or mixing cranberry and lingonberry juice (A6 [48]).

Another important aspect that was mentioned in most cranberry trials was the dosage of active ingredients administered to their patients (Table 1A). PAC is the main active ingredient unique to cranberries, which is reported to demonstrate an inhibitory effect against UPEC in the bladder [26]. Some trials attempted to characterise the PAC content in their commercial cranberry products, ranging from 2 to 112 mg. In addition, all except 1 trial revealed that their placebo drink contained ascorbic acid, which may have conferred protection onto the control group (A4, [46]). Hence, it is clear that characterising PAC content is very challenging and may even impact the final outcome of these clinical trials.

There were only two trials that utilised D-mannose (Table 1B). Interventions used either D-mannose powder (B1 [52]) or a tablet (B2 [53]), with 3 g of D-mannose/day as the maximum intake. Both trials of D-mannose used an antibiotic treatment as a control (Table 1B). For trials that examined the effectiveness of a combination of cranberry and D-mannose (Table 1C), trial C2 [55] used a capsule called Lactoflorene containing *lactobacillus paracasei* LC11, cranberry and D-mannose comparative to no prophylaxis as the control group. Trial C1 [54] used uticilin, which combines D-mannose, cranberry, bearberry and *olea europaea*, inulin, orthosiphon and *lactobacillus acidophilus*. The patients in the control group of this trial were not given prophylaxis but were advised to consume 1.5 L of water/day. 

Of the five NSAID trials (Table 1D), four trials (D2 [56], D3 [57], D4 [58] and D5 [59]) compared this to an antibiotic treatment, with one using an herbal product (*uva ursi*) as a comparison (D1 [77]). Different NSAID treatments and dosages were utilised in these trials. The dosage for NSAIDs varied from 200 mg aceclofenac (D5 [59]) to 1.2 g of ibuprofen (D1 [77], D2 [56], D4 [58]) and 1.8 mg ibuprofen (D3 [57]). As a comparison, 3 g of fosfomycin in D2 [56], 600 mg of pivmecillinam in D3 [57] and 500 mg of ciprofloxacin D4 [58] were used in these trials. Trial D1 [77] compared an NSAID to 3.6 g of *uva ursi*, and trial D5 [59] utilised a combination treatment of 200 mg cefpodoxime and 200 mg aceclofenac.

### 2.4. Effectiveness of Nonantibiotic Agents in Preventing UTI

One of the first criteria required in determining the effectiveness of nonantibiotic agents against UTIs is to decide how to measure success. One of the complicating factors in conducting this systematic literature review was that there is little agreement in terms of the measures of success used in the various studies (Table 2).

Sixteen of the trials including A1 [41], A2 [44], A3 [45], A4 [46], A5 [47], A6 [48], A7 [49], A8 [42], A9 [43], A10 [33], A11 [50], B1 [52], B2 [53], C1 [54], C2 [55] and D4 [58] measured the number of UTIs experienced by patients during the study period (Table 3). If there was a statistically significant reduction in the number of UTIs, this was deemed successful by the authors. Six trials were interested in time to first UTI (A1 [41], A2 [44], A3 [45], A4 [46], A10 [33], B1 [52]). Eleven trials calculated the percentage of patients who were free of UTI during the study period as a measure of success (A1 [41], A2 [44], A3 [45], A6 [48], A7 [49], A8 [42], A10 [33], A11 [50], A12 [51], C1 [54], C2 [55]). Six studies used the time to first UTI as an indication of success (A1 [41], A2 [44], A3 [45], A4 [46], A10 [33], B1 [52]), and one study determined the duration of UTI (A5 [47]). The five trials evaluating the effectiveness of NSAIDs used a symptom score [56,57,58,59,77]. For the studies examining the effectiveness of cranberry, D-mannose or a combination of these therapies, most trials studied the number of total UTIs and time to the first outcome.

The criteria for determining the effectiveness of treatment were further complicated by the varied criteria used by different trials to diagnose a UTI. There is disagreement internationally about the threshold that should be used to diagnose UTI. In 1992, the Infectious Disease Society of America (IDSA) published guidelines for the diagnosis and treatment of UTI [78] proposing a cut-off of >10^3^ CFU/mL for acute uncomplicated cystitis in women. This was reinforced by the 2009 guidelines from the European Association of Urology recommending > 10^3^ CFU/mL as the threshold for diagnosis of acute uncomplicated cystitis in women or, in the case of complicated UTI, >10^5^ CFU/mL [79]. Despite this, many studies, continue to use >10^5^ CFU/mL as their threshold for diagnosis of UTI [80,81,82] and the most recent joint ICS/IUGA consensus document endorses a threshold of >10^5^ CFU/mL for the diagnosis of UTI [83]. 

The global uncertainty about the most suitable cut-off for the diagnosis of UTI is visible in the data analysed for this study. For this review, the diagnostic criteria accepted by the authors were considered appropriate in terms of diagnosis of UTI. The studies evaluating the effectiveness of nonantibiotic agents against UTI defined as either greater than >10^3^ CFU/mL (*n* = 10, A1 [41], A2 [44], A8 [42], A9 [43], A11 [50], A12 [51], B1 [52], C1 [54], C2 [55] and D3 [57]) or >10^5^ CFU/mL (*n* = 6, A3 [45], A4 [46], A6 [48], A7 [49], A10 [33], B2 [53]). Two of the trials defined a UTI as >10^2^ CFU/mL (D2 [56], D4 [58]) and one trial defined UTIs as >10^4^ CFU/mL (D5 [71 Ko]). Two trials (A5 [47] and D1 [77]) did not measure the UTI threshold. A limitation of these two studies is the lack of scientific rigour that was applied to the diagnosis of UTI.

In comparison, most of the NSAID trials analysed symptom reduction. One of the most common symptoms of uncomplicated UTIs experienced by women is pain, which is a driving factor of UTI-associated voiding frequency [8,40]. Clinical trials evaluating the effectiveness of NSAID typically used a pain scale to characterise if there was a significant reduction in symptoms (D1 [77], D2 [56], D3 [57], D4 [58], D5 [59]). For the NSAID trials assessing effectiveness against the symptoms associated with UTI, the investigators developed their own pain scale to assess how effective the product was in alleviating UTI symptoms (Table 2). However, a symptom scale is rather subjective as pain tolerance may vary amongst patients. Hence, these discrepancies may have impacted the final trial outcomes. 

Regardless of the varied measures of success used, 50% of the cranberry trials reported success in terms of reducing the number of UTIs (Table 3A). Five studies (A1 [41], A2 [44], A4 [46], A5 [47] and A6 [48]) demonstrated a reduction in the number of UTI between the cranberry and control groups.

Three cranberry trials demonstrated a statistically significant difference in the time to the first UTI compared to the control group (A1 [41], A3 [45] and A4 [46]). However, this same result was not seen in two other cranberry trials (A2 [44] and trial A10 [33]). Trial A5 [47] reported a significantly shortened duration of UTI episodes in the cranberry groups (Table 3A). There were only two trials that utilised D-mannose, of which both trials were successful in reducing the number of UTIs (Table 3B). Both trials also demonstrated a reduced time to the first UTI in the D-mannose treatment compared to antibiotic groups (Table 3B). Hence, both trials reported low-level evidence of the effectiveness of D-mannose in preventing UTIs.

For combination products, both studies reported a reduction in the overall number of UTIs with lowered UTI-free days (Table 3C). Additionally, trial C1 [54] reported symptom reduction in the groups consuming the nonantibiotic product (data not shown in Table 3C). Of all the interventions, a combination of non-antibiotic products seemed to produce more consistent results in either reducing the number of symptomatic UTIs or treating symptom burden in women (A3 [45], A6 [48], A10 [33], C1 [54], C2 [55]). There is inadequate experimental evidence in the literature assessing the mechanism of action of combination treatments in treating bacterial infections. However, Ranfaing et al. recently demonstrated that cranberry and propolis combined generated a decreased expression of genes involved in adhesion, motility, biofilm formation and increased genes involved in iron metabolism and stress response [71]. Hence, with this limited in vitro evidence, it is plausible that combination treatments could potentiate a preventative effect in women with UTIs.

Amongst all the trials included in this study, eight studies compared a nonantibiotic treatment to an antibiotic (A10 [33], A11 [50], B1 [52], B2 [53], D2 [56], D3 [57], D4 [58] and D5 [59]). Only the trials of D-mannose (Table 3B, B1 [52], B2 [53]), revealed a significant difference in reducing the number of symptomatic UTIs in patients. Therefore, D-mannose or a combination of D-mannose with antibiotic treatment seemed to be the most beneficial in reducing the number of UTI. However, blinded-randomised trials with a significantly larger sample size are required to confirm these results.

Of the six NSAID studies (Table 3D), D2 [56] which compared ibuprofen to fosfomycin, demonstrated a significantly lower number of UTIs beyond the 2 weeks of ibuprofen administration. Despite this, ibuprofen treatment (D2 [56], D3 [57]) was associated with higher rates of hospitalisation and higher symptom burden, respectively. Trial D4 [58] did not report a statistically significant difference in the number of UTIs or symptom burden after treatment. However, trial D5 [59] reported faster symptom resolution in patients taking a combination of an NSAID (aceclofenac) and an antibiotic treatment (cepodoxime) at 1.5 days.

### 2.5. Side Effects

Another important outcome that was addressed in the trials was the side effects. A trial can only be deemed successful if there is a significant reduction in the outcomes analysed in conjunction with no burdening side effects. Cranberry seemed to have minimal impact on women. The main side effects experienced in cranberry groups were minor GI issues such as constipation, heartburn, loose stool, vaginal (itching, dryness) and migraines (Table 3A). There were also complaints in relation to reflux or bitter taste (A5 [48] and A7 [49]) since cranberry juice is typically very acidic (pH < 2.5), making it unpalatable even with sweetener for some patients [34]. Similarly, patients in trial A6 [48] reported stopping taking cranberry after a period of time due to its bitter taste, regardless of its effectiveness. 

No adverse events were reported in D-mannose trials (Table 3B,C). Only trial B1 [52] addressed women experiencing diarrhoea, nausea, headache, skin rash and vaginal burning for those taking an antibiotic treatment. Hence, D-mannose seems to be a relatively safe product for patients with r-UTI to consume. The use of NSAIDs typically resulted in a non-significant, but noticeable increase in pyelonephritis cases (Table 3D). This is most likely because NSAIDs only treat the UTI-associated symptoms but do not eradicate the actual bacterial infection [29].

Another important result that the researchers investigated was the predominant bacteria causing the UTIs in these women. More than 50% of the cranberry trials reported the initial causative organism at recruitment, which was *E. coli* (Table 3A). Both trials in the D-mannose and the two trials that investigated a combination of cranberry and D-mannose reported that the causative organism at recruitment was *E. coli* (Table 3B,C). Four of the six trials evaluating NSAIDs’ similarly also reported *E. coli* at recruitment (Table 3D).

Only trial B2 [53] and trial A9 [43], studying D-mannose and cranberry, respectively, observed the causative bacterial agent throughout the entire clinical trial period. *Enterococcus faecalis* was the second most prevalent strain. Following this, *Klebsiella pneumonia*, *Proteus mirabilis* and *Streptococcus agalactiae* were also reported. The majority of the literature reports on the mechanism of action for cranberry and D-mannose have focused on their effectiveness against *E. coli*. However, based on the results of this review, there seems to be no correlation between the success of cranberry in preventing or treating UTIs and whether the patient had an *E. coli* infection or not. This is in contrast to D-mannose. All of the studies using D-mannose (either alone or in combination) were seen to be successful, and all of the women in these trials were found to have an *E. coli* infection at recruitment, suggesting that D-mannose (alone or in combination) was successful in treating or preventing *E. coli* infection.

### 2.6. Limitations of Clinical Trial Data

The results of the trials examining the effectiveness of cranberry in preventing recurrent UTI produced conflicting results, with 50% of trials showing effectiveness and the other 50% being ineffective (Table 3A). This may be due to the small sample size used in most of these trials, meaning that the studies are not sufficiently powered to detect differences between the treatment groups (only 42% of cranberry trials reported being appropriately powered, Table 1A). This could also be owing to our very limited knowledge of the metabolic route in humans of the key active ingredients of cranberry, A-type PACs [84].

Some have proposed that PACs are inactive in vivo, since they are too large to be absorbed as intact molecules in the gastrointestinal tract and therefore the reported active ingredient in cranberry may not even make it to the urinary tract [85]. Comparatively, at least 90% of ingested D-mannose is efficiently absorbed in the upper intestine and is rapidly excreted from the bloodstream [86]. This difference in the metabolic pathways for these agents could potentially explain the discrepancies in trial outcomes between D-mannose and cranberry products.

Apart from the issues relating to the metabolism and availability of PAC, another issue relies on how different companies manufacture their cranberry products. A study of different commercially obtained cranberry products revealed that 20% contained 36 mg PACs/day, but actually contained up to 205 μg/g of procyanidin A2 [87]. Interestingly, anthocyanin retention in cranberry juice is typically less than 50% due to the various stages of food processing which leads to a substantial loss in phytochemicals [88]. This, therefore, indicates the lack of PAC standardisation and incongruency between global and individual compound analysis of cranberry products. Hence, this potentially explains the inconsistent results in utilising cranberry prophylaxis for uncomplicated cystitis in these trials. Overall, we cannot confirm if pharmacological deficiencies with cranberry, smaller sample sizes or poor PAC standardisation are responsible for producing such conflicting results. To overcome this, larger and randomised trials with proper PAC standardisation are required to confirm this.

Another factor that may have influenced these results is the amount of fluid intake in patients included in these trials. Patients with UTIs are commonly advised to increase fluid intake with a meta-analysis concluding that increased fluid intake leads to a statistically significant reduction in the number of people with recurrent UTIs within a 6-month period. The studies reviewed here did not report changes in participant fluid intake, which could be another contributor to varying results amongst the patients. More studies should include this criterion when screening their patients for these clinical trials [89].

Another limitation of these trials is the use of different antibiotics as the comparator of interest. This lack of consistency makes it challenging to compare the effectiveness of non-antibiotic agents against standard antibiotic therapy.

### 2.7. Limitations of This Review

A limitation of this systematic review is that it focused on human clinical trials and did not include experimental animal studies. Patients with complicated UTI were also excluded from this review. We also did not include pregnant women, men or children, as the majority of the trials recruited adult non-pregnant women. However, this was also an advantage of this study as it helped narrow the scope of papers to analyse for a comprehensive discussion on whether these nonantibiotic treatments could prevent or treat uncomplicated UTIs in women. In addition, in this study, the diversity of these trials, in terms of design, dosage, duration and outcome measures, made performing a meta-analysis a non-feasible option.

## 3. Conclusions

Overall, we have reviewed 21 trials investigating the outcome of different nonantibiotic treatments in preventing or treating UTIs in women. D-mannose or a combination of treatments seemed to be more effective in either preventing or treating UTIs. However, there were several limitations in these studies, including small sample sizes, therefore emerging as underpowered studies, conducting unblinded trials and using different definitions of a UTI. Future studies should investigate further into combination treatments with larger, blinded randomised trials.

The evidence for the efficacy of cranberry is inconclusive since 50% of the trials included were successful, with several factors influencing this, such as poor trial design and PAC standardisation. Although D-mannose was successful at reducing the number of UTIs, there were insufficient trials with a sufficient sample size to validate this result leading to a conclusion that there was low-level evidence of the effectiveness of D-mannose. NSAIDs also did not seem to be as effective for the symptomatic treatment of UTI. However, there may be an opportunity for combining antibiotic treatments with a nonantibiotic remedy to perhaps shorten the duration of antibiotic exposure. There is also potential for optimising these naturally occurring compounds to generate a higher efficacy. As an example, the characterisation of the D-mannose structure has resulted in the development of several synthetic mannosides [73], which have displayed a higher affinity to the FimH ligand compared to D-mannose [90]. Different synthetic mannosides have also been shown to prevent UPEC adhesion to cultured cells even better than D-mannose [67,72,91]. However, no trials have assessed the efficacy of synthetic mannosides within a human clinical trial. Overall, larger, randomised controlled samples are required to further understand whether these nonantibiotic products could play a role in preventing or treating UTIs in women.

## 4. Materials and Methods

### 4.1. Search Strategy

This systematic review was registered with Prospero, registration ID CRD42021297037. A comprehensive literature search of clinical trials was performed with a librarian using search strategies, keywords and standardised terms for urinary tract infection and for the three nonantibiotic treatments. Databases of PubMed, Embase and Scopus were searched with search terms pertaining to UTIs, including “urinary tract infection” OR “cystitis” OR “bacteriuria”. Search terms for cranberry included “proanthocyanidins” OR “*vaccinium macrocarpon*” OR “phytotherapy” OR “cranberry” OR “cranberry extract” OR “cranberry juice” OR “cranberry capsules”. Search terms for D-mannose included “mannosides” OR “D-mannose”. For NSAIDs, keywords included “non-steroidal anti-inflammatory drugs”, OR “diclofenac” OR “naproxen”. Titles and abstracts were screened for potentially eligible full texts, and human studies published in English were included in this study.

### 4.2. Inclusion and Exclusion Criteria

Eligible studies were clinical trials conducted in humans, undertaken with a focus on the prevention or treatment of UTIs using the three specified nonantibiotic treatments (cranberry, mannose and NSAIDs). The trials only included adult women (≥18 years old) with uncomplicated cystitis. Uncomplicated cystitis was defined as an infection occurring within the urinary tract with no prior instrumentation or comorbidities. Patients with complicated UTIs occurring in the presence of a functional or structural abnormality including instrumentation (e.g., an indwelling urethral catheter) or significant medical or surgical issues were excluded from this review. Studies focused on UTI in pregnant women were also excluded. Similarly, studies focused on asymptomatic bacteriuria, classified as persistent bacterial colonisation in the urinary tract without an individual showing symptoms, were also excluded from this paper. Additionally, animal experimental studies were excluded from this review.

## Figures and Tables

**Table 1:** Summary of study design for clinical trials of cranberry (**A**), D-mannose (**B**), combination cranberry and D-Mannose (**C**) and NSAIDS (**D**).

**A: Study Design of Cranberry Trials**					
**Name Year**	**Study Design**	**Focus**	**Intervention**	**Mean Age**	Sample Size (Reported Power *)
**A1. Koradia, 2019 [41]**	Randomised, double-blind, placebo-controlled, parallel group pilot clinical trial; 6 months duration	rUTI Prevention	Group 1: BKPro-Cyan (36 mg PAC) Group 2: Placebo capsule 2×/day	Group 1:34.6 (SD:9.6) Group 2:34.8 (SD:10.1)	90
**A2. Maki, 2016 [44]**	Randomised, double-blind, placebo-controlled, multicentre clinical trial; 6 months duration	rUTI Prevention	Group 1: 8-oz (240-mL) cranberry (DMAC-method I: 41.1 ± 7.1 DMAC-method II: 119 ± 16.9) Group 2: Placebo beverage (240 mL)	Group 1:40.9 (SEM:1.1) Group 2:41.0 (SEM:1.0)	373 (Yes)
**A3. Bruyère, 2019 [45]**	Randomised double-blinded, controlled study; 6 months duration	rUTI Prevention	Group 1: 2× cranberry-propolis-zinc (DUAB) /day Group 2: 2× placebo capsules/day;	Group 1:53.0 (SD:17.4) Group 2:53.0 (SD:19.2)	85 (Yes)
**A4. Vostalova, 2015 [46]**	Single centre, randomised, double-blind placebo-controlled trial 7 months duration	rUTI Prevention	Group 1: Cranberry: 500 mg CFP (2× 250 mg CFP capsules) 1×/day (2.8 mg PAC) Group 2: Placebo capsule	Group 1:35.6 (SD:12.9) Group 2:38.0 (SD:13.4)	182 (Yes)
**A5. Ledda, 2015 [47]**	Pilot registry study 2 months duration	rUTI Prevention	Group 1: Cranberry: 500 mg CFP (2× 250 mg CFP capsules) 1×/day (2.8 mg PAC) Group 2: Placebo;	Group 1:39 (SD:4) Group 2:39 (SD:3)	44
**A6. Kontiokari, 2001 [48]**	Open, randomised controlled 12 month follow up trial	rUTI Prevention	Group 1: 50 mL cranberry-lingonberry juice concentrate daily, 6 months Group 2: 100 mL of lactobacillus drink 5 days /week for 12 months Group 3: No intervention	Group 1:32 (SD:9.8) Group 2:30 (SD:11.8) Group 3:29 (SD:10.5)	150 (No)
**A7. Stothers, 2002 [49]**	Randomised, controlled, double-blind clinical trial; 12 months duration	rUTI Prevention	Group 1: Placebo arm: 2×/day + 250 mL placebo juice 3×/day Group 2: Tablet arm: 1 cranberry 2×/day + 250 mL placebo juice 3×/day Group 3: Juice arm: 250 mL of unsweetened cranberry juice 3×/day + 1 placebo 2×/day;	Average (all groups) 42	150
**A8. Stapleton, 2012 [42]**	Randomised double-blinded, controlled study 6 months duration	rUTI Prevention	Group 1: 4 oz of cranberry juice daily Group 2: 8 oz of cranberry juice daily Group 3: Placebo juice	Group 1:25.3 (SD:6.6) Group 2:26.4 (SD:6.5)	176 (No)
**A9. Barbosa-Cesnik, 2011** **[43]**	Prospective, randomised, double-blind comparison 6 months duration	rUTI Prevention	Group 1: 8 oz (240 mL) of 27% low-calorie cranberry juice cocktail 2×/day (112 mg PAC) Group 2: 8 oz of placebo juice 2×/day	Group 1:21.2 (SD:3.4) Group 2:21.2 (SD:3.5)	319 (Yes)
**A10. Gbinigie, 2021 [33]**	Open-label feasibility randomised parallel group trial 7 days duration	Acute cystitis Treatment	Group 1: Immediate antibiotics Group 2: First-line antibiotics + cranberry capsules Group 3: Only cranberry capsules 2× cranberry 2×/day	Group 1:52.4 (SD:21.4) Group 2:52.4 (22.4) Group 3:40.8(14.0)	46 (No)
**A11. Beerepoot, 2011 [50]**	Randomised double-blind, double dummy noninferiority trial 12 months duration	rUTI, prevention	Group 1: Trimethoprim-sulfamethoxazole (TMP-SMX), 480 mg/ day Group 2: Cranberry capsules, 500 mg 2×/day (9.1 mg PAC)	Median (IQR): Group 1:36.1 (27–46) Group 2:34.8 (23–44)	221 (No)
**A12. Babar, 2021 [51]**	Randomised, controlled, double-blind clinical trial 5.5 months duration	rUTI, prevention	Group 1: Cranberry PAC extract Urophenol (2 × 18.5 mg/day) Group 2: Control low dose (2 × 1 mg/ day	Group 1:32.5 (SD:14.2) Group 2:27.2 (SD:8.8)	145
**B. Study design of D-mannose trials**					
**Name Year**	**Study Design**	**Focus**	**Intervention**	**Mean age**	Sample size (Reported Powered *)
**B1. Kranjčec, 2014 [52]**	Prospective, randomised controlled study 6 months duration	rUTI, Prevention	Group 1: 2 g D-mannose powder in 200 mL of water daily Group 2: 50 mg Nitrofurantoin/day Group 3: No prophylaxis	Median (age range) Group 1: 49 (38–56) Group 2: 48 (29–58) Group 3: 52 (38–56)	308 (Yes)
**B2. Porru, 2014 [53]**	Randomised cross-over trial 6 months duration	rUTI, Prevention	Group 1: TMP-SMX 160 mg/800 mg–2×/day for 5 days, then single dose at night for 1 wk/month for next 23 wks Group 2: D-mannose 1 g 3×/day, every 8 h for 2 weeks, then 1 g–2×/day for 22 weeks Cross-over point at 24 wks	Average (all groups) 42	60
**C. Study design of combination cranberry and D-mannose trials**					
**Name Year**	**Study Design**	**Focus**	**Intervention**	**Mean age**	Sample size (Reported Powered *)
**C1. Mainini, 2020 [54]**	Prospective cohort study 10 days for 12 months	rUTI, Prevention	Group 1: Used compound (Uticlin containing D-mannose, cranberry, bearberry, Olea europaea), inulin, Orthosiphon and Lactobacillus acidophilus) Group 2: No prophylaxis; Advised to consume 1.5 L water/ day	Group 1:53.0 (SD:5.8) Group 2:54.1 (SD:4.5)	94
**C2. Murina, 2021 [55]**	Single-centre study 3 months duration	rUTI, Prevention	Group 1: Lactoflorene Cist 1×/day for 10 days/month (*Lactobacillus paracasei* LC11, cranberry and D-mannose) Group 2: Lactoflorene Cist 1×/day for 90 days Group 3: No treatment	Mean (range) Group 1:38.2 (20–43) Group 2:39.4 (22–45) Group 3:38.4 (21–41)	55 (Yes)
**D. Study design of NSAID trials**					
**Name Year**	**Study Design**	**Focus**	**Intervention**	**Mean age**	Sample size (Reported Powered*)
**D1. Moore, 2019 [55]**	Factorial randomised trial 3–5 days duration	Acute cystitis, Treatment	Group 1: Uva-ursi + ibuprofen Group 2: Placebo + ibuprofen Group 3: Uva-ursi + no ibuprofen Group 4: Placebo + no ibuprofen, Dosage: Uva-ursi: daily dose of 3600 mg; Ibuprofen: daily dose of 1200 mg	Group 1:45.5 (SD:15.2) Group 2:39.9 (SD:15.5) Group 3:44.6 (SD:16.1) Group 4:44.8 (SD:14.3)	382 (No)
**D2 Gágyor, 2015 [56]**	Double blind randomised multicentre comparative effectiveness trial; 3 days duration	Acute cystitis, Treatment	Group 1: Fosfomycin 3 g Group 2: Ibuprofen 3 × 400mg	Group 1:37.3 (SD:14.3) Group 2:37.3 (SD:14.6)	494 (Yes)
**D3. Vik, 2018 [57]**	Double-blind, randomised, parallel group, multicentre non-inferiority trial; 3 days duration	Acute cystitis, Treatment	Group 1: 600 mg Ibuprofen Group 2: 200 mg pivmecillinam 3×/day	Group 1:28.1 (SD:8.6) Group 2:28.5 (SD:10.2)	383 (Yes)
**D4. Bleidorn, 2010 [58]**	Double-blind, randomised controlled pilot trial 3 days duration	Acute cystitis, Treatment	Group 1: Ibuprofen 3 × 400 mg oral Group 2: Ciprofloxacin 2 × 250 mg (+1 placebo)	Group 1:44.5 Group 2:43.7	80 (No)
**D5. Ko, 2018 [59]**	Double-blind, randomised controlled pilot trial 3 days duration	Acute cystitis, Treatment	Group 1: Cepodoxime (100 mg 2×/day) Group 2: Cepodoxime (100 mg) + aceclofenac (100 mg) 2×/day	Average (all groups) 49.9 (SD:13.5)	55 (No)

* Reported Power—Conclusions regarding power of the study are based on that reported by the original authors of the study.

**Table 2:** Measures of success in prevention or treatment of UTI.

Measure	Definition	References
Number of UTI	Statistically significant reduction in the number of UTIs in treatment versus placebo group.	[41,42,43,44,45,46,47,48,49,50,51,52,53,54,55,58]
Time to first UTI	Statistically significant reduction in the time to first UTI in treatment versus placebo group.	[33,41,44,45,46,52]
% Of patients UTI free	Statistically significant difference susceptible patients who did not acquire a UTI during this study period.	[33,41,42,44,45,48,49,50,51,54,55]
Duration of UTI	Statistically significant difference in the total time frame patients experienced the UTI for up until symptom resolution	[47]
Symptom scores	Significant UTI-associated symptom relief experienced in patients after taking treatment	[56,57,58,59,77]

**Table 3:** Outcomes of clinical trials of cranberry (**A**), D-Mannose (**B**), combination cranberry and D-Mannose (**C**) and NSAIDs (**D**).

A. Outcomes of Cranberry Trials				
Name Year	UTI Threshold (CFU/mL) Bacteria Isolated	Outcome Reported	Side Effects Reported	Reported Success *
**A1. Koradia, 2019 [41]**	≥ 10^3^ N/A	**No of UTIs:** BKProCyan vs. placebo: 4 (9.1%) vs. 33.3% (*p* = 0.005) **Time to first UTI:** Median time: 174 vs. 90 days (*p* = 0.001) **UTI free:** BKPro-Cyan group vs. placebo group (90.9 vs. 66.7%) **Duration of UTI:**N/A **Symptom Scores:**N/A	3 (6.8%) had abdominal distention (*n* = 1) and diarrhoea (*n* = 2) All reported TEAEs were mild in severity, considered related to the study drug and resolved without corrective treatment	Yes
**A2. Maki, 2016 [44]**	≥ 10^3^ *E. coli*	**No of UTIs:** Cranberry vs. placebo: 39 vs. 67 (*p* = 0.016) **Time to first UTI:** Cranberry vs. placebo: 33 (17.8%) vs. 50 (26.6%) first UTI with pyuria (*p* = 0.131) **UTI free:** Cranberry vs. Placebo: 33 subjects (17.8%) vs. 50 (26.6%) (HR: 0.67; 95% CI: 0.43, 1.05; *p* = 0.078) **Duration of UTI:**N/A **Symptom Scores:**N/A	GI issues: 3 (1.6%) with nausea (*p* = 0.044) Headache [cranberry group: *n* = 16 (8.6%); sinusitis [cranberry group: *n* = 10 (5.4%)] and upper respiratory infection [cranberry group: *n* = 13 (7.0%); All serious AEs unlikely related to cranberry product	Yes
**A3. Bruyère, 2019 [45]**	≥ 10^5^ *E. coli*	**No of UTIs:** Frequency of cystitis in first 3 mo: 0.7 ± 1.1 propolis + cranberry group vs. 1.3 ± 1.1 placebo (*p* = 0.0257), but not in the first 6 months Total cystitis episodes within first 3 mo: 1.4 ± 1.0 vs. 2.0 ± 1.0 (*p* = 0.0680) **Time to first UTI:** 69.9 ± 45.8 days vs. 43.3 ± 45.9 (*p* = 0.0258) **UTI free:** 47.1% in the propolis and cranberry vs. 36.1% in the placebo group (*p* = 0.3527) **Duration of UTI:**N/A **Symptom Scores:**N/A	3 events: Sigmoid diverticulitis, acute pyelonephritis and hallux valgus corrective surgery. All unrelated to treatment	Yes
**A4. Vostalova, 2015 [46]**	≥ 10^5^ *E. coli*	**No of UTIs:** Average UTIs/subject: 0.12 (10/83 vs. 0.32 (30/93) (*p* = 0.03) **>**1 UTI: 9/83 (10.84%) in cranberry vs. 24/93 (25.81%) in placebo (*p* = 0.04) **Time to first UTI:** 133 days in the cranberry group vs. 65 days in placebo group (*p* = 0.04) **UTI free:**N/A **Duration of UTI:**N/A **Symptom of scores:**N/A	None reported	Yes
**A5. Ledda, 2015 [47]**	N/A N/A	**No of UTIs:** 73.3% reduction in freq. of UTI (*p* < 0.05) 15.4% reduction in control group (*p* = 0.012) **Time to first UTI:**N/A **UTI free:** N/A **Duration of UTIs:** 2.5 ± 1.3 vs. 3.6 ± 1.7 days (*p* < 0.05) **Symptom scores:**N/A	3 (13.6%) in cranberry group required medical consultation for UTI symptoms (*p* < 0.05). However, no AEs reported	Yes
**A6. Kontiokari, 2001 [48]**	≥ 10^5^ *E. coli*	**No of UTIs:** Difference between cranberry and control] (0.36 UTIs /person year) (95% CI 0.03–0.68, *p* = 0.03) [Cranberry: 0.45: Lactobacillus: 0.91: Control: 0.81 **Time to first UTI:** N/A **UTI free:** 8 (16%), 19 (39%) and 18 (36%): 20% reduction in cranberry vs. control (95% CI: 3% to 36%, *p* = 0.023) **Duration of UTI:**N/A **Symptom scores:** N/A	No AEs reported except for occasional complaint of the bitter taste of cranberry juice	Yes
**A7. Stothers, 2002 [49]**	≥ 10^5^ N/A	**No of UTIs:** Mean no. UTIs was 0.72 in placebo group, 0.30 in juice group (*p* < 0.05) and 0.39 in tablet group (*p* < 0.05) **Time to first UTI:** N/A **UTI free:** Placebo: 16 (32%), Juice: 10 (20%, *p* < 0.05) and tablet 9 (18%, *p* < 0.05) **Duration of UTI:**N/A **Symptom scores:**N/A	Juice: reflux; tablet: mild nausea, increased frequency of bowel movements	No
**A8. Stapleton, 2012 [42]**	≥ 10^3^ *E. coli*	**No of UTIs:** Cranberry vs. placebo: (33/120, 27.5%) vs. placebo group (17/56; 30.4%) **Time to first UTI:** N/A **UTI free:** Not significantly different between groups (*p* = 0.41) **Duration of UTI:**N/A **Symptom scores:**N/A	3 discontinued due to GI symptoms: including constipation, heartburn, vaginal (itching, dryness) and other (migraine) symptoms	No
**A9. Barbosa-Cesnik, 2011 [43]**	≥ 10^3^ *E. coli*	**No of UTIs:** Cranberry vs. placebo: vs. cumulative incidence rate: 19.3% vs. 14.6% (*p* = 0.21) **Time to first UTI:** N/A **UTI free:** N/A **Duration of UTI:**N/A **Symptom scores:**N/A	SAEs unrelated to treatment	No
**A10. Gbinigie, 2021 [33]**	**>**10^5^ N/A	**No of UTIs:** N/A **Time to first UTI:** Reduced in group 2 (adjusted mean difference: 0.6; 95%CI: −4.0 to 5.3) and increased in group 3 (adjusted mean diff: 7.9; 95%CI 2.6–13.2) vs. control **UTI free:** Reduced in group 2 (adjusted HR 1.7. 95% CI: 0.7 to 4.1) but increased in group 3 (adjusted HR 0.6; 95% CI: 0.2 to 1.4), compared with controls **Duration of symptoms:**N/A **Symptom scores:**N/A	7 total events: Immediate antibiotics and immediate cranberry: suprapubic pain, dysuria, backache, cloudy urine. Immediate cranberry and delayed antibiotics: feeling unwell, ache in lower back, backache, stomach-ache, headache and nausea	No
**A11. Beerepoot, 2011 [50]**	≥ 10^3^ *E. Coli*	**No of UTIs:** TMP-SMX vs. Cranberry: After 3 mo: 0.5 (95% CI:0.3–0.7) and 0.7 (95% CI: 0.4–0.9) (*p* = 0.30) After 12 mo: 1.8 (95% CI: 0.8–2.7) vs. 4.0 (2.3–5.6) (*p* = 0.02) **Time to first UTI:** N/A **UTI free:** TMP-SMX vs. cranberry: Median time: 8 mo vs. 4 mo (*p* = 0.03) **>**1 UTI TMP-SMX vs. cranberry: After 3 months: 32.1 (19.7.42.5) and 36.9 (22.9–48.3) (*p* = 0.75) **Duration of UTI:**N/A **Symptom scores:**N/A	None	No
**A12. Babar, 2021 [51]**	≥ 10^3^ N/A	**No of UTIs:** High dose vs. low PAC: 45 vs. 59 **Time to first** UTI: N/A **UTI free:** Median high dose vs. low PAC: 24.0 wks vs. 16.6 **Duration of UTI:**N/A **Symptom scores:**N/A	No serious AEs. Only one person reported dyspepsia, leading to a discontinuation	No
**B. Outcomes of D-mannose trials**				
**Name Year**	**UTI threshold (CFU/mL)**	**Outcome reported**	**Side effects reported**	**Reported** Success *
**B1. Kranjčec, 2014 [52]**	≥ 10^3^ *E. coli*	**No of UTIs:** D-mannose: 15 (14.6%) Nitrofurantoin:21 (20.4%) No prophylaxis:62 (60.8%) (*p* < 0.001 comparing prophylaxis to control) **Time to first UTI:** Median time: Mannose: 43 days (IQR: 15–50) vs. Nitrofurantoin: 24 days (IQR: 15–36) vs. no prophylaxis: 28 (20–42) (*p* = 0.12) **UTI free:**N/A **Duration of UTI:**N/A **Symptom scores:**N/A	7.8% (*p* < 0.001) Diarrhoea (8/103)	Yes
**B2. Porru, 2014 [53]**	≥ 10^5^ *E. coli*	**No of UTIs:** Of the 60 women, 45 (75%) had 1 recurrence, 10/60 (16.6%) had 2 recurrences, 5/60 (8.3%) had no r-UTI **Time to first UTI:** 52.7 days (D-mannose) vs. 200 days (antibiotics) (*p* < 0.0001) **Time to first UTI:** N/A **UTI free:**N/A **Duration of symptoms:**N/A **Symptom scores:**N/A	None reported	Yes
**C. Outcomes of combination cranberry and D-mannose trials**				
**Name Year**	**UTI threshold (CFU/mL)**	**Outcome reported**	**Side effects reported**	Reported Success *
**C1. Mainini, 2020 [54]**	≥ 10^3^ *E. coli*	**No of UTIs:** Group 1 (active): 6-month follow-up: 8/48 (*p* = 0.0057) 12-month follow-up: 11/48 (*p* = 0.0005) Group 2 (placebo): 6-month follow-up: 3/46 (p = 0.2418) 12-month follow-up: 4/46 (*p* = 0.1168 **Time to first UTI:**N/A **UTI free:** Patients without r-UTI: Group 1 (active): 6-month follow-up: 8/48 (*p* = 0.0057) 12 months: 11/48 (*p* = 0.0005)Group 2 (placebo): 6-month follow-up: 3/46 (*p* = 0.2418) 12 months:4/46 (*p* = 0.1168) **Duration of symptoms:**N/A **Symptom scores:**N/A	None reported	Yes
**C2. Murina, 2021 [55]**	≥ 10^3^ *E. coli*	**No of UTIs:** Group 1:16% Group 2:15.5% Group 3:52.9% (*p* < 0.01) **UTI Free:** Day 90: Group 1:87.7% Group 2:84.9%. Group 3:42% Day 150: Group 1:65.8% Group 2:68.8% Group 3:36.9% **UTI free:**N/A **Duration of symptoms:**N/A **Symptom scores:**N/A	None reported	Yes
**D. Outcomes of NSAID trials**				
**Name Year**	**UTI threshold (CFU/mL)**	**Outcome reported**	**Side effects reported**	**Reported** Success *
**D1. Moore, 2019 [77]**	N/A N/A	**No of UTIs:** N/A **Time to first UTI:**N/A **UTI free:**N/A **Duration of symptoms:**N/A **Symptom score:** Freq. symptom severity on days 2–4, showed no difference in symptom severity between factorial groups	No UUT infections	Yes
**D2. Gágyor, 2015 [56]**	>10^2^ *E. coli*	**No of UTIs**: N/A After day 14: Fosfomycin: 11% vs. 6% Ibuprofen (*p* = 0.049) **Time to first UTI:**N/A **UTI free:**N/A **Duration of symptoms:**N/A **Symptom score:** Higher symptom burden on days 0–7 in ibuprofen group	4 in ibuprofen needed hospital admission; 1 GI haemorrhage likely due to Ibuprofen	No
**D3. Vik, 2018 [57]**	≥10^3^ *E. coli*	**No of UTIs:** N/A **Time to first UTI:**N/A **UTI free:**N/A **Duration of symptoms:**N/A **Symptom score:** Mean symptom sum score: 2.3 for Ibuprofen and 0.7 for pivmecillinam, estimated difference of 1.6 (95% CI 0.8–2.4)	7 pyelonephritis cases in ibuprofen group	No
**D4. Bleidorn, 2010 [58]**	>10^2^ *E. coli*	**No of UTIs:** Follow up: Day 28, 1 ciprofloxacin patient and 2 ibuprofen patients (*p* = 1.0) reported relapse **Time to first UTI:**N/A **UTI free:**N/A **Duration of UTI:**N/A **Symptom course (SD):** ibuprofen vs. ciprofloxacin. Day 7: 0.7 (1.26) vs. 0.6 (0.86) (*p* = 0.816)	Mostly GI disorders and upper respiratory tract infections, headaches likely unrelated to treatment	No
**D5. Ko, 2018 [59]**	≥10^4^ *E. coli*	**No of UTIs:** N/A **Time to first UTI:**N/A **UTI free:**N/A **Duration of UTI:**N/A **Symptom score:** Faster symptom resolution in combination therapy at 1.5 days (*p* = 0.035)	N/A	No

* Reported success: the decision about the success of the trial was based on the conclusions reported by the author of the original studies.

## Data Availability

Not applicable.
